# Plasma cytokine profiles in systemic sclerosis: associations with autoantibody subsets and clinical manifestations

**DOI:** 10.1186/ar2821

**Published:** 2009-10-02

**Authors:** Pravitt Gourh, Frank C Arnett, Shervin Assassi, Filemon K Tan, Mei Huang, Laura Diekman, Maureen D Mayes, John D Reveille, Sandeep K Agarwal

**Affiliations:** 1 Division of Rheumatology and Clinical Immunogenetics, Department of Internal Medicine, University of Texas Health Science Center at Houston, 6431 Fannin M.S.B. 5.278, Houston, TX 77030, USA

## Abstract

**Introduction:**

Systemic sclerosis (SSc) (scleroderma) is a complex autoimmune disease that clinically manifests as progressive fibrosis of the skin and internal organs. Anti-centromere antibodies (ACAs), anti-topoisomerase antibodies (ATAs), and anti-RNA polymerase III antibodies (ARAs) are three mutually exclusive SSc-associated autoantibodies that correlate with distinct clinical subsets characterized by extent of cutaneous involvement and pattern of organ involvement. The current report sought to determine whether plasma cytokine profiles differ in SSc patients grouped according to these SSc-associated autoantibody subsets.

**Methods:**

Plasma from 444 SSc patients and 216 healthy controls was obtained from the Scleroderma Family Registry and University of Texas Rheumatology Division. Patients were classified according to the presence of ACAs, ATAs, ARAs, or none of the above (antibody-negative). Levels of 13 cytokines were determined using multiplex assays.

**Results:**

Compared with females, healthy control males had higher plasma levels of IL-2 (*P *= 0.008), IL-5 (*P *= 0.01) and IL-8 (*P *= 0.01). In addition, in controls, IL-6 (*P *= 0.02) and IL-17 (*P *= 0.01) levels increased with advancing age. After adjusting for age and gender, SSc patients had higher circulating levels of TNFα (*P *< 0.0001), IL-6 (*P *< 0.0001), and IFNγ (*P *= 0.05) and lower IL-17 (*P *= 0.0005) and IL-23 (*P *= 0.014). Additional analyses demonstrated that disease duration also influenced these cytokine profiles. IL-6 was elevated in ATA-positive and ARA-positive patients, but not in ACA-positive patients. IL-8 was uniquely increased in the ATA-positive subset while both ATA-positive and ACA-positive subsets had elevated IFNγ and IL-10. IL-5 was only significantly increased in the ACA-positive subset. Lastly, patients with interstitial lung disease had elevated IL-6 and patients with pulmonary hypertension had elevated IL-6 and IL-13.

**Conclusions:**

Plasma cytokine profiles differ in SSc patients based on the presence of SSc-associated autoantibodies. Plasma cytokine profiles in SSc patients may also be affected by disease duration and the pattern of internal organ involvement.

## Introduction

Systemic sclerosis (SSc) (scleroderma) is a chronic, multisystem autoimmune disease clinically characterized by progressive fibrosis of the skin and internal organs. Pathologically, SSc exhibits three cardinal features: inflammation and autoimmunity, vasculopathy, and excessive extracellular matrix production and deposition. How the disease process is triggered remains to be established, but current paradigms point towards immune dysregulation as a central process in the pathogenesis of SSc.

Multiple lines of evidence support the importance of immune dysregulation in the pathogenesis of SSc. Skin biopsies of early scleroderma skin demonstrate perivascular infiltrates of mononuclear inflammatory cells, including CD4^+ ^T cells, which produce cytokines and chemokines that induce tissue damage, recruit additional inflammatory cells, and promote extracellular matrix production and fibrosis [1]. Whole genome gene expression profiling of peripheral blood has demonstrated the presence of a type-I interferon signature in SSc [2]. There have been conflicting reports in the literature regarding the role of T cells and the T-helper type 1 (Th1)/T-helper type 2 (Th2) cytokine balance in SSc. Some studies support Th1 activation in the peripheral blood with production of IFNγ, while others predict a preferential involvement of Th2 cells in SSc with increased levels of IL-4 and IL-13 [3-5]. Lastly, several reports have demonstrated increased circulating levels of cytokines in plasma of patients with SSc compared with controls with conflicting results [4,6-11]. These conflicting results may be due to the samples being collected in different stages of the disease process. Alternatively, these conflicting results could reflect the heterogeneity amongst SSc patients.

The presence of multiple SSc-associated autoantibodies has been well described [12-15]. Interestingly, the SSc-associated autoantibodies correlate with distinct clinical subsets characterized by the extent of cutaneous involvement and the pattern of organ involvement [15]. For example, pulmonary arterial hypertension is more common in patients with anti-centromere antibodies (ACAs), pulmonary fibrosis is more common in patients with anti-topoisomerase antibodies (ATAs), and scleroderma renal crisis is more common in patients with anti-RNA polymerase III antibodies (ARAs) [15]. Whether the clinical differences observed in these autoantibody subsets also reflect differences in immune dysregulation is not known.

In the current report, a comprehensive panel of cytokines was assessed in a large cohort of SSc patients and controls to determine whether SSc patient have differences in plasma cytokines and whether these profiles correlate with autoantibody subsets of SSc.

## Materials and methods

### Systemic sclerosis patients and controls

Patients and unrelated controls were selected from the Scleroderma Family Registry and DNA Repository and University of Texas Rheumatology Division, dating from 1986 to present [16]. All SSc patients fulfilled American College of Rheumatology preliminary criteria for disease classification [17] or had at least three of the five features of CREST (calcinosis, Raynaud's phenomenon, esophageal dysfunction, sclerodactyly, and telangiectasias). All SSc patients were classified based on the presence of scleroderma-associated autoantibodies including ACAs, ATAs, and ARAs or the absence of these three antibodies (Ab-Neg). SSc patients negative for antinuclear antibodies were excluded from this study. From these groups, a total of 444 SSc patients were randomly chosen from a cohort of 665 SSc patients. Two hundred and sixteen healthy controls were also randomly selected. Samples used in the study were obtained at the earliest time point available.

The patients were classified as having limited or diffuse cutaneous SSc according to published criteria [18]. SSc-associated pulmonary fibrosis was defined as the presence of typical findings on chest high-resolution computerized tomography, regular chest computerized tomography or radiograph, or a restrictive pattern on pulmonary function testing. Pulmonary hypertension was defined as estimated peak right ventricular systolic pressure ≥ 40 mmHg on echocardiography or pulmonary arterial systolic pressure ≥ 40 mmHg by right heart catheterization. Scleroderma renal crisis was characterized by the presence of new-onset accelerated systemic hypertension with evidence of renal impairment. Myositis was defined as inflammatory myositis referenced in the patient's chart or as objective muscle weakness and elevated creatine kinase levels.

All study subjects provided written informed consent and the study was approved by the institutional review board of the University of Texas Health Science Center at Houston.

### Autoantibody analysis

Sera were tested for antinuclear antibodies using indirect immunofluorescence with HE*p*-2 cells as the antigen substrate (Antibodies Inc., Davis, CA, USA). ACAs were determined by their distinctive indirect immunofluorescence pattern on HE*p*-2 cells. Autoantibodies to topoisomerase I were determined by passive immunodiffusion against calf thymus extract (Inova Diagnostics, San Diego, CA, USA). ARAs were determined by enzyme-linked immunoassay (MBL Co. Ltd, Nagoya, Japan) using a cutoff value defined as 2.5 standard deviations above the mean of 40 controls.

### Enzyme-linked immunosorbent assay

Plasma was collected in ethylenediamine tetraacetic acid blood collection tubes and stored at -80°C for bulk analysis. Cytokine ELISAs were performed using electrochemiluminescent multiplex assays (Meso Scale Discovery, Gaithersburg, MD, USA) to determine the plasma levels of 13 cytokines (IFNγ, TNFα, IL-1β, IL-2, IL-4, IL-5, IL-6, IL-8, IL-10, IL-12p70, IL-13, IL-17 and IL-23) [19]. Calibration curves were prepared in the supplied assay diluents for human serum, with a range of 2,500 to 1.2 pg/ml. Cytokine concentrations were determined with MSD Workbench 3.0 software (Meso Scale Discovery, Gaithersburg, MD, USA), using curve fit models (log- log or four-parameter log-logistic).

### Statistical analysis

Statistical analyses were performed using SAS 9.1.3 software (SAS Institute Inc., Cary, NC, USA). Cytokine data were log-transformed due to the non-normal distribution of plasma cytokines in both the healthy controls and the SSc population [20]. The cytokine values were compared between two groups using an unpaired Student's *t *test. Logistic regression analysis was used to compare plasma cytokine levels when controlling for age and gender. Association of plasma cytokines with clinical manifestations of SSc was performed using logistic regression with adjustment for age and gender.

## Results

### Demographics

The cohort consisted of 216 healthy controls and 444 scleroderma patients of similar ages (Table 1). Of the scleroderma patients, 241 patients had limited SSc and 200 patients had diffuse SSc. All patients were antinuclear antibody-positive, and SSc-associated antibodies were present in 330 patients: 109 patients with ACAs, 112 patients with ATAs, and 109 patients with ARAs. One hundred and fourteen patients were negative for all three SSc-associated antibodies (Ab-Neg). Furthermore, no patients had more than one of the above SSc-associated antibodies present.

**Table 1:** Demographics and clinical data of the cohort

	Controls (n = 216)	Scleroderma patients (n = 444)
Age (years)	51 ± 14 years	53 ± 12.1
Gender		
Male	117 (54%)	56 (13%)
Female	99 (46%)	388 (87%)
Scleroderma phenotype		
Limited		241 (54%)
Diffuse		200 (45%)
Disease duration (years)		6.9 ± 0.3
Systemic sclerosis-association autoantibodies		
Anti-centromere		109 (25%)
Anti-topoisomerase		112 (25%)
Anti-RNA polymerase III		109 (25%)

Data presented as mean ± standard deviation or *n *(%).

The mean disease duration for all SSc patients was 6.9 years from the time of first non-Raynaud's phenomenon manifestation. ACA-positive patients had the longest disease duration, with a mean duration of 9.3 years. ATA-positive patients had a mean disease duration of 8.0 years, and ARA-positive patients and Ab-Neg patients had disease durations of 5.4 years and 4.9 years, respectively. The differences in disease duration in patients grouped according to SSc-associated antibodies were statistically significant (*P *< 0.0001 by analysis of variance).

### Age and gender changes in plasma cytokines in healthy controls

To determine whether plasma cytokines levels were affected by age and gender, plasma cytokine levels were determined on plasma from 216 healthy controls. Plasma cytokine levels were not normally distributed across the cohort of healthy controls, so the data were log_*n*_-transformed [20].

**Figure 1:**
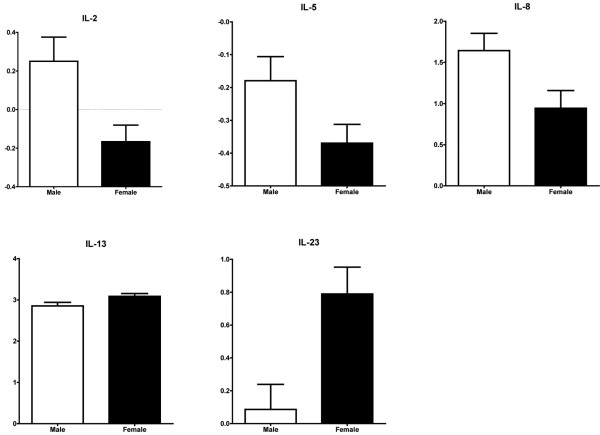
Gender effects on plasma cytokine levels.  Male healthy controls have higher circulating levels of IL-2 (*P *= 0.008), IL-5 (*P *= 0.01) and IL-8 (*P *= 0.01), and lower circulating levels of IL-13 (*P *= 0.03) and IL-23 (*P *= 0.006). Plasma cytokine levels (in pg/ml) were log_*n*_-transformed. Data presented as mean ± standard error of the mean.

Compared with females (n = 99), male healthy controls (n = 117) had higher circulating levels of IL-2 (*P *= 0.008), IL-5 (*P *= 0.01) and IL-8 (*P *= 0.01) (Figure 1). In contrast, male controls had lower circulating levels of IL-13 (*P *= 0.03) and IL-23 (*P *= 0.006). Male controls tended to have increased circulating levels of IL-1β, IL-4 and IL-10 (*P *< 0.10) but no significant differences were observed in circulating levels of TNFα, IFNγ, IL-6, IL-12p70, and IL-17.

**Figure 2:**
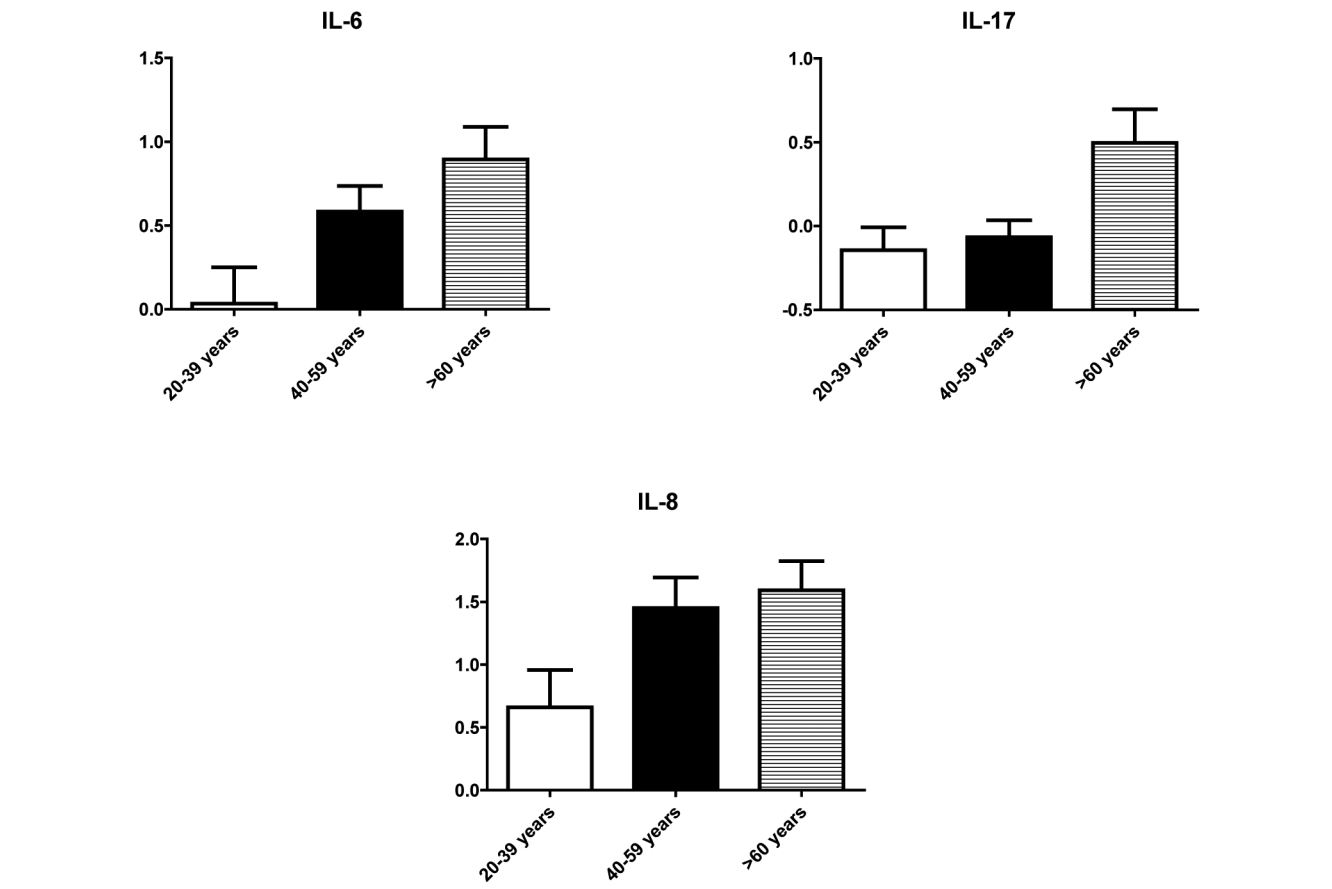
Age effects on plasma cytokine levels.  IL-6 (*P *= 0.02) and IL-17 (*P *= 0.01) levels increased with advancing age. There was also a trend for increasing levels of IL-8 with age (*P *= 0.07). Plasma cytokine levels (in pg/ml) were log_*n*_-transformed. Data presented as mean ± standard error of the mean.

To determine whether increasing age alters the circulating plasma cytokine levels, healthy controls were grouped according to age in 20-year intervals. As seen in Figure 2, IL-6 (*P *= 0.02) and IL-17 (*P *= 0.01) levels increased with advancing age. There was also a trend for increasing levels of IL-8 with age (*P *= 0.07). No changes were noted in IL-1β, IL-2, IL-4, IL-5, IL-10, IL-12p70, IL-13, IL-23, IFNγ, and TNFα.

Together these data demonstrate that circulating cytokine levels are different in healthy males and females and are dependent on age.

### Changes in plasma cytokines in scleroderma patients

Circulating cytokine levels were determined in plasma from SSc patients and were compared with those for control patients (Table 2). Compared with healthy control subjects, SSc patients had higher circulating levels of TNFα (*P *< 0.0001), IL-6 (*P *< 0.0001) and IL-13 (*P *= 0.05) but lower circulating levels of IL-17 (*P *= 0.0009) and IL-23 (*P *= 0.04). No differences were observed in circulating levels of IL-1β, IL-2, IL-4, IL-5, IL8, IL-10, and IFNγ.

Given the observation that age and gender can influence plasma cytokine levels in healthy controls, comparisons between scleroderma patients and healthy controls were performed adjusting for age and gender using logistic regression analysis (Table 2). After adjusting for age and gender, patients with SSc had higher circulating levels of TNFα and IL-6 and lower circulating levels of IL-17 and IL-23. In addition, SSc patients had higher circulating levels of IFNγ; however, the previous unadjusted change in plasma IL-13 was no longer significant.

**Table 2:** Plasma cytokine levels of healthy controls and scleroderma patients

	Healthy controls (n = 216)		Scleroderma patients (n = 444)		*P *value	
	
	pg/ml	log_*n *_cytokine	pg/ml	log_*n *_cytokine	Unpaired *t *test	Adjusted for age and gender
TNFα	8.9 ± 0.5	1.90 ± 0.06	14.1 ± 0.7	2.27 ± 0.05	<0.0001	<0.0001
IL1β	7.0 ± 1.2	0.36 ± 0.11	5.8 ± 0.8	0.38 ± 0.07	NS	NS
IL-6	5.2 ± 0.8	0.55 ± 0.11	9.7 ± 1.2	1.24 ± 0.07	<0.0001	<0.0001
IL-8	77.7 ± 20.1	1.32 ± 0.15	84.3 ± 13.8	1.58 ± 0.11	NS	NS
IL-2	2.8 ± 0.7	0.05 ± 0.08	2.4 ± 0.4	0.02 ± 0.05	NS	NS
IFNγ	2.9 ± 0.5	- 0.17 ± 0.08	8.2 ± 4.6	- 0.06 ± 0.06	NS	0.05
IL-12p70	16.2 ± 4.3	0.66 ± 0.12	53.2 ± 12.8	0.71 ± 0.09	NS	NS
IL-4	5.1 ± 0.8	0.38 ± 0.11	5.4 ± 1.2	0.32 ± 0.07	NS	NS
IL-5	1.2 ± 0.2	- 0.27 ± 0.05	11.1 ± 5.1	- 0.14 ± 0.06	NS	NS
IL-13	24.6 ± 1.7	2.97 ± 0.06	51.5 ± 12.0	3.12 ± 0.05	0.05	NS
IL-10	18.5 ± 11.6	0.62 ± 0.10	30.6 ± 10.0	0.83 ± 0.08	NS	0.07
IL-17	1.9 ± 0.2	0.04 ± 0.08	1.2 ± 0.1	- 0.22 ± 0.04	0.0009	0.0005
IL-23	4.7 ± 1.1	0.46 ± 0.12	3.7 ± 0.5	0.18 ± 0.07	0.04	0.014

Plasma cytokine levels are presented as both absolute levels (pg/ml) as well as natural log-transformed (log_*n *_cytokine) to normalize the data for analysis. Data presented as the mean ± standard error of the mean. *P *value determined either by unpaired *t *test or by logistic regression controlling for age and gender; NS, not significant with *P *> 0.05.

Together these data demonstrate that SSc patients have an increase in circulating levels of TNFα, IL-6, and IFNγ and a decrease in IL-17 and IL-23.

### Effect of disease duration on cytokine profiles in scleroderma patients

Immune dysregulation is commonly thought to be important in the early pathogenesis of SSc. Whether the cytokine alterations that might be observed early in the disease process persist as the disease progresses, however, is unclear. To determine whether disease duration influenced the patterns of plasma cytokine profiles, patients were grouped according to disease duration: 0 to 5 years (n = 196 patients), 5 to 10 years (n = 107 patients), and >10 years (n = 94 patients). The disease duration was not known for 47 SSc patients, and these patients were excluded from this analysis.

**Figure 3:**
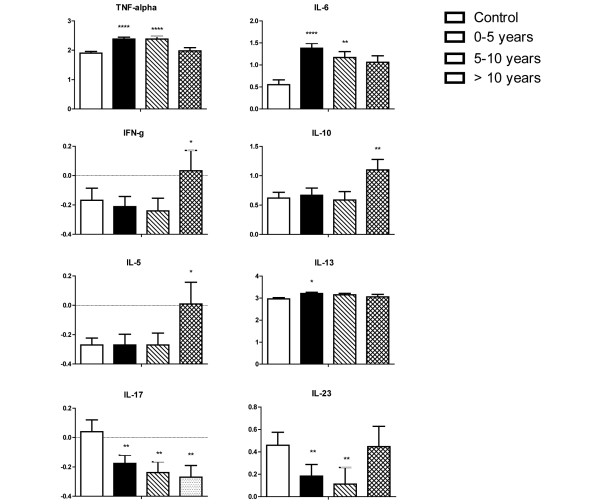
Effect of disease duration on plasma cytokine profiles in scleroderma patients compared with controls.  Plasma cytokine levels (in pg/ml) were log_*n*_-transformed. Data presented as mean ± standard error of the mean. *P < 0.05. **P < 0.01. ****P < 0.0001.

TNFα and IL-6 were significantly increased in SSc patients with a disease duration of 0 to 5 years (*P *< 0.0001 and *P *< 0.0001, respectively) and a duration of 5 to 10 years (*P *< 0.0001 and *P *= 0.007, respectively) compared with controls (Figure 3). TNFα and IL-6 levels were similar between controls and SSc patients with disease duration >10 years. Alterations in circulating levels of other cytokines became apparent after controlling for disease duration as well as age and gender. For example, circulating levels of IL-5, IL-10 and IFNγ were only elevated in patients with disease duration >10 years compared with healthy controls (*P *= 0.04, *P *= 0.008, and *P *= 0.03, respectively). In contrast, IL-13 was slightly increased in patients with disease duration <5 years (*P *= 0.03). Lastly, IL-17 levels remained decreased in patients independent of disease duration, but IL-23 levels were only decreased in SSc patients with disease duration of 0 to 5 years or 5 to 10 years compared with controls.

These data suggest that the circulating cytokine profiles are different in patients based on disease duration, and suggest that alterations in immune balance may change during different stages of SSc.

### Cytokine profiles of scleroderma patients based on the presence of scleroderma-associated autoantibodies

The presence of scleroderma-associated autoantibodies is associated with distinct clinical phenotypes of SSc [15]; however, it is not known whether these different subsets have different alterations in immune function. Comparisons of plasma cytokines were performed in each group based on the presence of scleroderma-associated autoantibodies (ACAs, ATAs, ARAs, Ab-Neg), controlling for age and gender using logistic regression analysis.

**Figure 4:**
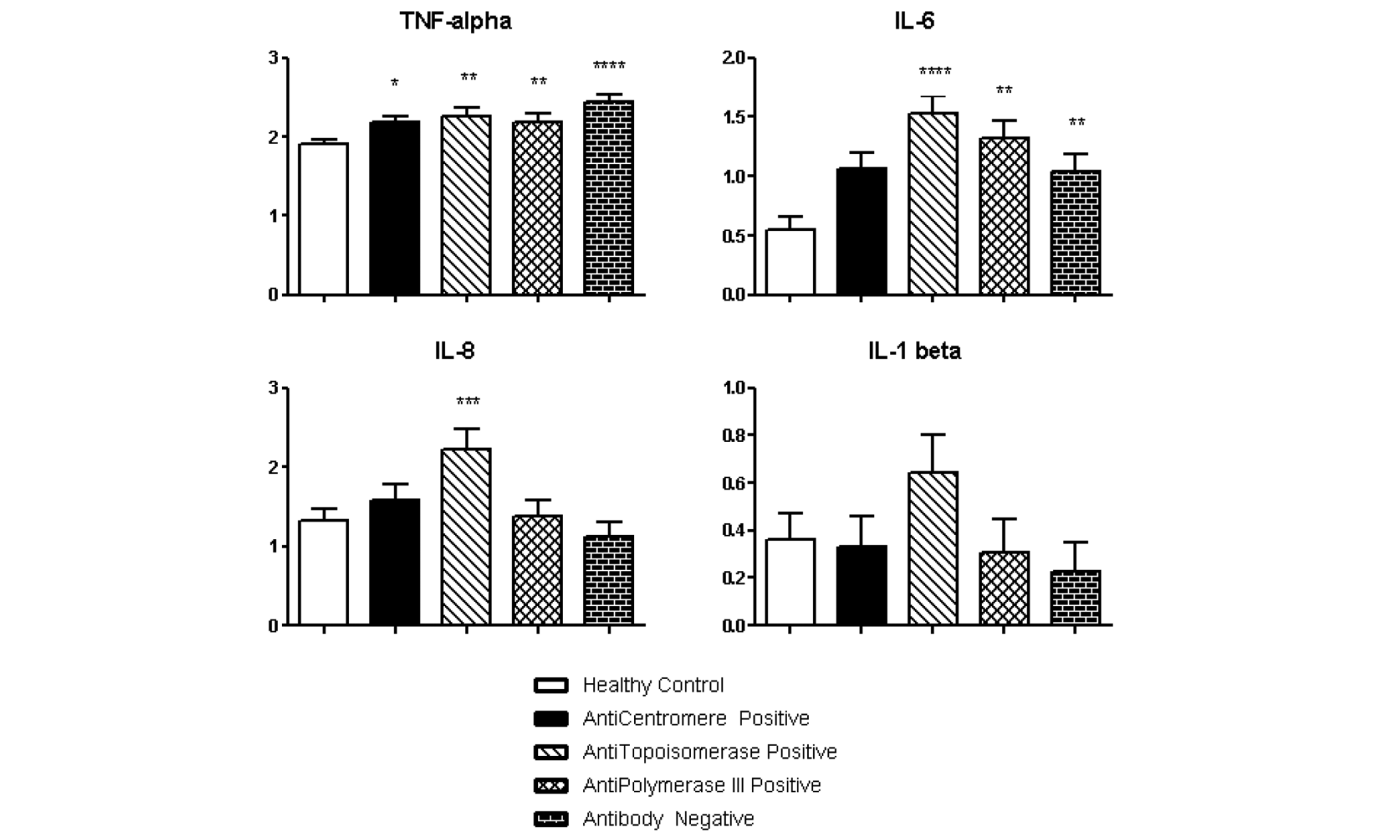
Four plasma cytokine profiles in systemic sclerosis-association autoantibody subsets of scleroderma patients compared with controls.  Plasma cytokine levels (in pg/ml) were log_*n*_-transformed. Data presented as mean ± standard error of the mean. *P < 0.05. **P < 0.01. ***P < 0.001. ****P < 0.0001.

**Figure 5:**
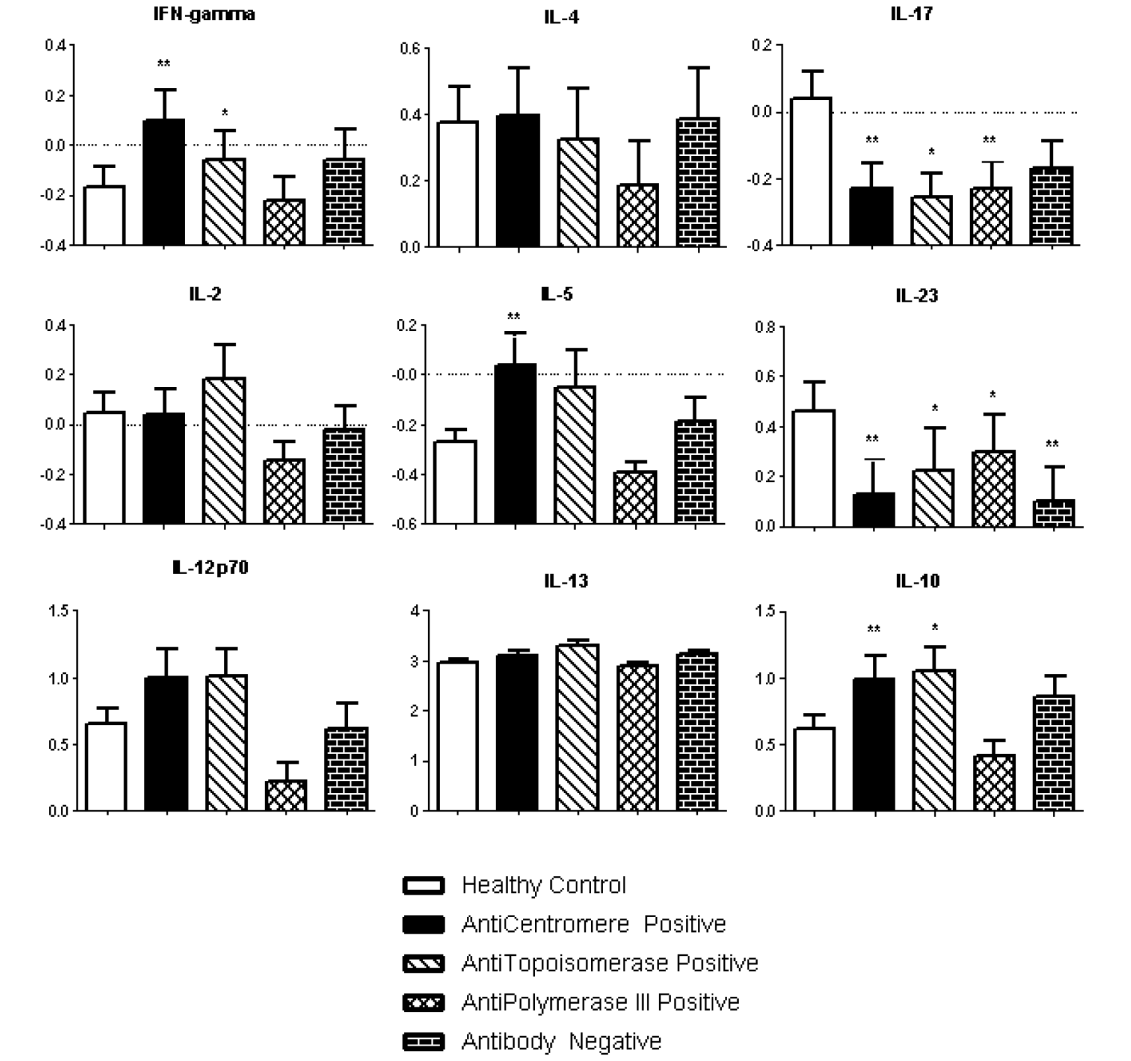
Nine plasma cytokine profiles in systemic sclerosis-association autoantibody subsets of scleroderma patients compared with controls.  Plasma cytokine levels (in pg/ml) were log_*n*_-transformed. Data presented as mean ± standard error of the mean. *P < 0.05. **P < 0.01.

As seen in Figures 4 and 5, all four groups of SSc patients had a significant increase in TNFα and a decrease in IL-23. The ATA-positive, ARA-positive and Ab-Neg subsets had a statistically significant increase in IL-6, which was not observed in the ACA-positive group. Furthermore, IL-17 was significantly decreased in all groups compared with controls, except in the Ab-Neg group. IL-8 was uniquely increased in the ATA-positive subset. Interestingly, both ATA-positive and ACA-positive subsets had an increase in IFNγ and IL-10, but IL-5 was only significantly increased in the ACA-positive subset. Lastly, the ARA-positive and Ab-Neg group did not have alterations in circulating IFNγ, IL-8 or IL-10.

Together these data suggest that the presence of SSc-associated autoantibodies may identify patients with different patterns of circulating cytokines and that different pathways of immune dysregulation may underlie the development of SSc.

### Association of cytokine profiles with clinical manifestations of scleroderma

To determine whether circulating plasma cytokine profiles were associated with clinical involvement in SSc, plasma cytokines in patients with and without interstitial lung disease, pulmonary hypertension, SSc renal crisis, Sjogren's syndrome/sicca symptoms, myositis or primary biliary cirrhosis were compared with controls, using logistic regression to control for age and gender (Table 3). Patients were excluded from this analysis if the specific status was not known.

**Table 3:** Plasma cytokine associations with clinical involvement of scleroderma

	Present	Absent	Unknown	Cytokine	Odds ratio	95% confidence interval	*P *value
Interstitial lung disease	120	196	128	IL-6	1.33	1.08 to 1.62	0.006
Pulmonary hypertension	58	255	131	IL-6	1.31	1.04 to 1.67	0.02
				IL-13	1.42	1.04 to 1.94	0.03
SSc renal crisis	20	294	130	TNFα	2.22	1.11 to 4.45	0.02
Sjogren's syndrome	118	199	127	IL-8	0.89	0.81 to 0.99	0.03
				IL-1β	0.75	0.63 to 0.88	0.0006
Myositis	17	297	130	None			
Primary biliary cirrhosis	5	309	140	IL-17	3.46	1.35 to 8.9	0.01

Cytokine levels were normalized using *log*_*n *_transformation. Logistic regression was used to compare cytokine levels to control subjects for gender and age and odd ratios reflect each cytokine as a continuous variable.

Patients with higher circulating IL-6 were more likely to have interstitial lung disease (odds ratio = 1.33, 95% confidence interval = 1.08 to 1.62). Patients with pulmonary hypertension were more likely to have higher IL-6 (odds ratio = 1.31, 95% confidence interval = 1.04 to 1.67) and IL-13 (odds ratio = 1.42, 95% confidence interval = 1.04 to 1.94). Patients with SSc renal crisis were more likely to have elevated TNFα levels (odds ratio = 2.22, 95% confidence interval = 1.11 to 4.45). Interestingly, patients with Sjogren's syndrome/sicca symptoms were more likely to have lower IL-8 (odds ratio = 0.89, 95% confidence interval = 0.81 to 0.99) and IL-1β (odds ratio = 0.75, 95% confidence interval = 0.63 to 0.88).

Finally, we performed a logistic regression analysis using the individual components of the Medsger Damage Index (general, peripheral vascular, skin score, joint/tendon, muscle, gastrointestinal tract, and heart) as a continuous variable[21]. Interestingly, IL-6 was associated with total skin scores (regression coefficient = 0.77; 95% confidence interval = 0.05 to 1.49) and IL-17 levels were associated with joint/tendon scores (regression coefficient = 0.27; 95% confidence interval = 0.10 to 0.43).

Together these data demonstrate that there are distinct differences in the plasma cytokine profiles in patients representing different clinical manifestations of SSc.

## Discussion

In the current report, the circulating plasma cytokine profile was determined in patients with SSc compared with controls using a large cross-sectional cohort of SSc patients and healthy controls. We observed that SSc patients have higher levels of TNFα, IL-6 and IFNγ, but lower levels of IL-17 and IL-23. We also observe that the disease duration and the presence of SSc autoantibodies have an influence on these cytokine profiles. Lastly, it was discerned that specific clinical manifestations of SSc, such as interstitial lung disease, pulmonary hypertension or SSc renal crisis, are also associated with alterations in distinct plasma cytokine levels.

Collectively, these data bring to light the complex immunopathogenesis of SSc and echo the clinical heterogeneity that is seen within SSc. The SSc-associated autoantibodies are clinically useful to risk-stratify patients for the systemic involvement of SSc. Accordingly, we observe that there are also differences in circulating cytokine levels when using these autoantibodies to classify SSc patients. For example, ATA-positive patients are the only subset with an increase in IL-8. ATA-positive and ACA-positive patients both have an increase in IFNγ and IL-10, while only ACA-positive patients present an increase in IL-5. Based on the current data, the ARA-positive subset appears to be a distinct subset. Similar to ATAs and ACAs, ARAs show an increase in TNFα and IL-6 as well as a decrease in IL-17 and IL-23. The ARA-positive subset, however, does not have alterations in the Th1/Th2 cytokines that are observed in the ATA-positive subset or the ACA-positive subset.

Alterations in plasma cytokine levels, including IL-6, TNFα, IL-10 and IL-4, have been reported by several groups in the past, with varying results [6-10]. The differences are probably due to the heterogeneity within SSc, differences in disease duration, differences in gender, disease activity, and small sample size of these studies. The current report utilizes the largest cohort of SSc patients and healthy controls to date. This enables controlling for variables such as gender and age, which we have shown can influence circulating cytokine levels even in healthy controls as well as in SSc patients. Similar to previous reports, we demonstrate an increase in circulating levels of IL-6 [6,7]. Interestingly, these changes appear to be less significant in patients with longer disease duration, although we cannot rule out a contribution of disease activity. IL-6 is a pleiomorphic cytokine produced by T cells, B cells, monocytes, endothelial cells, and fibroblasts, and is involved in regulating many cellular processes including multilineage blast cell colony formation, T-cell differentiation and fibroblast behavior [22]. IL-6 is therefore probably a key cytokine in the immunopathogenesis of SSc.

Scleroderma has often been considered a Th2 cytokine disease [3,23]. Th1 cytokines such as IFNγ have antifibrotic effects, while Th2 cytokines such as IL-4 and IL-13 have profibrotic effects [24]. Indeed, Th2 cells have been cloned from SSc skin with greater frequency than Th1 cells, although not exclusively [3]. Other reports have not, however, consistently demonstrated a Th2 cytokine profile in SSc patients [11,25,26]. In the current report, the ATA-positive and ACA-positive subsets have an increase in the Th1 cytokine IFNγ. Both subsets present an increase in the Th2 cytokine IL-5, but it is only statistically significant in the ACA-positive subset. These data do not point to a selective increase in Th2 cytokines. Additional studies using more sensitive measures of the Th1/Th2 cytokine balance are needed to better address this aspect.

T-helper type 17 cells have recently been implicated as key T cells in the pathogenesis of autoimmune diseases, such as multiple sclerosis, rheumatoid arthritis and ankylosing spondylitis [27-29]. IL-17 and IL-23 have been reported to be increased in the plasma of patients in two small Japanese cohorts of SSc patients [30,31]. In the current large cohort, when controlling for age, gender, and autoantibody status, we observed a decrease in circulating IL-17 and IL-23, especially in patients with shorter disease duration. We cannot definitively explain these differences but possible explanations are the sample sizes of the cohorts and the differences in genetic background of Japanese versus the current cohort of North American Caucasians, which may influence the cytokine profile. Given the potential importance of the T-helper type 17 pathway in autoimmune diseases, future efforts should focus on delineating the role of T-helper type 17 cells in SSc immunopathogenesis.

A final observation of potential interest is the association of circulating cytokines with systemic manifestations of SSc. Similar to prior reports, we observed an association of IL-6 with interstitial lung disease [6]. We also noted that IL-6 and IL-13 levels were increased in patients with pulmonary hypertension, and that TNFα levels were associated with the presence of renal crisis. While alterations in cytokine levels were associated with clinical manifestations of SSc, prospective studies are needed to determine whether measuring these cytokine levels in patients would be helpful in predicting the development of these detrimental clinical manifestations.

The current report has several limitations that should be acknowledged. While the plasma samples were obtained from a large cohort of SSc patients and healthy controls, accurate data for immunosuppressive medications were not available at the time of the present publication. Another limitation is the cross-sectional design of the current study. The data reported herein identify disease duration as a factor that influences cytokine profiles. This might be particularly relevant with regards to the ACA-positive group, which has longer disease duration than the other autoantibody groups. A prospective study with sequential plasma samples would be beneficial to better understand the immune changes that are associated with the development of SSc as well as the progression of the disease and disease activity. It should also be noted that the SSc-associated autoantibody group (Ab-Neg) remains a heterogeneous group of patients - as defined by the presence of other autoantibodies such as anti-fibrillarin, anti-PM-Scl or anti-Th/To [32] - and whether these less common subsets also have differences in the plasma cytokine profiles remains to be determined.

## Conclusions

SSc is associated with alterations in circulating plasma cytokines. These alterations are influenced by gender, age, disease duration and the presence of specific SSc-associated autoantibodies. These results highlight the complex immunopathogenesis of SSc and point to several potential targets that could be considered for monitoring disease progression and treatment of SSc.

## Abbreviations

Ab-Neg: antibody-negative; ACA: anti-centromere antibody; ARA: anti-RNA polymerase antibody; ATA: anti-topoisomerase antibody; ELISA: enzyme-linked immunosorbent assay; IFN: interferon; IL: interleukin; SSc: systemic sclerosis; Th1: T-helper type 1; Th2: T-helper type 2; TNF: tumor necrosis factor.

## Competing interests

The authors declare that they have no competing interests.

## Authors' contributions

PG, FCA, FKT, MDM, JDR and SKA designed the project. PG, SA, MH, LD, MDM, JDR and SKA acquired the data. PG, FCA, SA, FKT, MH, LD and SKA analyzed the data. All authors read and approved the final manuscript.
